# Functional Variation in RGS12 Should Not Preclude Methylphenidate Use in Bipolar Disorder with Established Mood Stabilization: Preclinical Evidence

**DOI:** 10.3390/ijms262411993

**Published:** 2025-12-12

**Authors:** Percy S. Agogo-Mawuli, Joshua D. Gross, Vincent Setola, Bryan J. Gall, David P. Siderovski

**Affiliations:** 1 Department of Pharmacology and Neuroscience, College of Biomedical & Translational Sciences, University of North Texas Health Science Center, Fort Worth, TX 76107, USA; 2 Departments of Nutritional Sciences and Biobehavioral Health, Huck Life Sciences Institute, Pennsylvania State University, University Park, PA 27710, USA; jdg6119@psu.edu; 3 Department of Behavioral Medicine & Psychiatry, WVU School of Medicine, Rockefeller Neuroscience Institute, West Virginia University, Morgantown, WV 26506, USA; vssetola@hsc.wvu.edu; 4 Department of Neuroscience, WVU School of Medicine, Rockefeller Neuroscience Institute, West Virginia University, Morgantown, WV 26506, USA; 5 Variant Science Team, NeoGenomics, Fort Myers, FL 33912, USA

**Keywords:** attention-deficit/hyperactivity disorder, bipolar disorder, methylphenidate, psychostimulant-induced locomotion, regulator of G protein signaling-12 (RGS12)

## Abstract

Our goal in this study was to determine whether functional variation in the human *RGS12* gene influences behavioral responses to psychostimulants such as methylphenidate, thereby informing whether such genetic findings should affect the clinical use of this central nervous system (CNS)-stimulating agent in bipolar disorder (BD) patients with comorbid attention-deficit/hyperactivity disorder (ADHD). The use of psychostimulants for ADHD in BD remains controversial due to concerns about mood destabilization, although recent systematic reviews indicate that methylphenidates and amphetamines can be safe and effective when used with mood stabilizers. RGS12, a striatally enriched regulator of κ-opioid receptor signaling and dopamine transporter (DAT) function, has been implicated in altered dopaminergic responses to psychostimulants. A recently characterized R59Q reduction-of-function mutation within RGS12 has been associated with familial bipolar disorder, further highlighting its potential relevance to mood and psychostimulant responsiveness. *Rgs12*-deficient mice were evaluated for behavioral responses to methylphenidate (i.e., locomotor hyperactivity) and compared with responses to dopamine transporter-dependent stimulants. *Rgs12* deficiency was seen to reduce hyperlocomotion with amphetamine, and with methamphetamine but not with methylphenidate, which was instead observed to elicit normal hyperlocomotor responses across all doses. Methylphenidate responsiveness remains intact despite the loss of RGS12 function, suggesting that *RGS12* functional variation in the human condition should not contraindicate methylphenidate use in mood-stabilized BD/ADHD comorbidity.

## 1. Introduction

Bipolar disorder (BD) is a chronic illness characterized by recurrent episodes of depression and hypomanic or manic episodes, whereas attention-deficit/hyperactivity disorder (ADHD) is a neurodevelopmental condition marked by persistent inattention, impulsivity, and hyperactivity. Although each disorder is traditionally viewed as distinct in terms of age of onset, symptomatology, and clinical course, their co-occurrence is strikingly common [1]. A recent meta-analysis estimated that up to one in six individuals with BD also meet the diagnostic criteria for ADHD [2]. Clinical studies further show that BD patients with comorbid ADHD tend to have an earlier age at onset, more frequent depressive and hypomanic or manic episodes, and a more chronic, disabling course of illness than BD patients without ADHD [3,4]. This high rate of co-occurrence and its clinical consequences underscore the public health importance of understanding how ADHD symptoms and BD interact—including how one might optimally treat ADHD in BD without destabilizing mood.

From a neurobiological perspective, a convergence of dopaminergic dysfunction offers a plausible shared substrate for both disorders. In ADHD, decades of imaging and pharmacologic data implicate the dysregulation of dopamine transporter (DAT) function and broader catecholaminergic signaling as central to the manifestations of inattention, impulsivity, and hyperactivity (e.g., refs. [5,6]). In BD, accumulating evidence points to aberrant dopamine–system regulation, particularly in brain circuits governing reward, motivation, and mood stability, as a mechanism contributing to depressive and hypomanic or manic episodes and vulnerability to hypomanic or manic episodes [1,7]. Thus, genetic variation in protein functions that control dopamine release and reuptake or neurotransmitter receptor signaling represent attractive candidates to explain the overlap between BD and ADHD. Such shared neurobiological underpinnings lend biological plausibility to the hypothesis that genetic variation affecting dopamine-regulatory pathways could influence both disease risk and response to psychostimulant treatment in individuals with comorbid BD and ADHD.

Using psychostimulants to treat ADHD in individuals with BD remains a subject of debate within psychiatric research and clinical practice. Concerns center on the potential for stimulants, such as amphetamine and methylphenidate, to precipitate mood destabilization, particularly by triggering hypomanic or manic episodes. However, recent systematic reviews have challenged the assumption that psychostimulants invariably induce mood dysregulation in BD, instead suggesting that they may be both efficacious and safe when used judiciously alongside mood stabilizers.

A recent systematic review and meta-analysis conducted by Canadian colleagues [8] evaluated the risk of hypomanic or manic episode recurrence in BD patients prescribed psychostimulants or other pro-cognitive medications. Their findings suggest that contrary to prevailing clinical caution, psychostimulant use in euthymic or depressed BD patients does not significantly increase mania scores on the Young Mania Rating Scale (YMRS). Their review included published data from randomized controlled trials (RCTs) and prospective studies, synthesizing evidence from 27 studies, five of which were included in the formal meta-analysis. Pooled data (*n* = 1653) revealed no statistically significant increase in mania scores among patients treated with psychostimulants compared to placebo, and qualitative synthesis further suggested a limited risk of medication-induced manic episodes.

The conclusions offered by Chiorean et al. [8] align with findings from prior studies of the safety and efficacy of psychostimulants in bipolar disorder when used alongside mood stabilizers. A Swedish registry-based study by Viktorin et al. [9] found that methylphenidate did not significantly increase the risk of manic switching in BD patients who were concurrently treated with mood stabilizers, whereas those not receiving such agents exhibited an elevated risk of manic episodes. Similarly, randomized controlled trials in pediatric BD populations have demonstrated that methylphenidate and mixed amphetamine salts can effectively improve ADHD symptoms without exacerbating manic episodes, provided that mood stabilization is achieved before stimulant initiation. Findling et al. [10] found that methylphenidate significantly reduced the burden of ADHD symptoms in children and adolescents with BD without inducing mood destabilization, while Scheffer et al. [11] found that amphetamine treatment following mood stabilization with delayed-release valproate resulted in significant ADHD symptom improvement without triggering manic symptoms. In further support, Zeni et al. [12] reported observing no significant increase in manic symptoms when methylphenidate was administered adjunctively with aripiprazole in pediatric BD patients with comorbid ADHD.

Similar to the recent Canadian review [8], the International Society for Bipolar Disorders (ISBD) Targeting Cognition Task Force conducted a systematic review [13] examining both established and off-label ADHD drug therapies for cognitive impairment and ADHD symptoms in BD. Across 17 studies analyzed (*n* = 2136), the Task Force found no increased risk of hypomanic or manic episodes in BD patients treated with psychostimulants, provided they were concurrently receiving mood-stabilizing medications. Notably, the Task Force’s report emphasized that the risk of manic episodes was greater in patients not receiving mood stabilizers, a nuance that Chiorean et al. did not explicitly highlight [8]. The Task Force’s report also found that methylphenidate and mixed amphetamine salts were particularly effective in improving attention and ADHD symptoms, though not global cognition, in BD patients [13].

Collectively, the findings and reviews mentioned above have significant clinical implications in suggesting that the carefully selected BD patient with comorbid ADHD (a comorbidity that is not a rare occurrence [1,2,14]) may benefit from psychostimulants without undue risk of mood destabilization. Could the field turn now to (re)establishing genetic biomarkers as guides for this “careful selection” for individualized treatment decisions? Prior advances in psychiatric genetics have already unveiled complex polygenic architectures underlying BD and ADHD (e.g., refs. [15,16,17,18]), with growing emphases on their overlap (e.g., refs. [19,20] reinforcing long-standing recognition of shared neurobiological substrates [21,22,23]) and on risk-associated genetic variants that may directly influence pathophysiology and/or pharmacologic response [24,25,26,27,28,29,30].

In this latter context of genetic biomarkers, *RGS12* (Regulator of G protein Signaling 12) has recently been implicated as a candidate susceptibility gene for both BD and ADHD. RGS12 encodes a multi-domain scaffolding protein with a unique, central enzymatic activity (the “RGS-box”; [31,32]) for inactivating neurotransmitter signaling transacted by G protein-coupled receptors (GPCRs); we have recently established a central role for the RGS-box activity of RGS12 in inhibiting κ-opioid receptor (KOR)-mediated influences on dopamine release and reuptake in the CNS [33,34]—pathways critically linked to both disorders (e.g., refs. [22,23]). Contemporaneously, Forstner et al. [35], using a whole-exome sequencing approach, identified a rare, functionally significant variant of RGS12, NM_001394154.1:c.176G>A (p.Arg59Gln/R59Q), in individuals with high-penetrance familial BD. Forstner et al. hypothesized that the R59Q variation impaired protein–protein interactions essential for synaptic regulation, potentially altering neural circuits involved in mood regulation and attentional control. Given the high degree of phenotypic overlap between BD and ADHD, the discovery of this inherited genetic variant has raised an intriguing question regarding the role of RGS12 in modulating responses to psychostimulants in BD patients with comorbid ADHD.

Recent mechanistic studies in mice point to RGS12 acting as a critical intracellular scaffold and GTPase-accelerating protein that links G-protein-coupled receptor (GPCR) signaling to presynaptic control of monoamine transporter function [33,34], extending even beyond dopamine and its presynaptic dopamine transporter (DAT) protein [36]. Initially, *Rgs12* knockout mice were seen to exhibit marked alterations in KOR-dependent signaling within the ventral striatum: signal transduction alterations that lead to dysregulated DAT activity and blunted behavioral responses to dopamine transporter-dependent psychostimulants such as cocaine and amphetamine [33,34]. Electrochemical recordings using fast-scan cyclic voltammetry further revealed that these dopaminergic abnormalities are directly observable within the nucleus accumbens, where RGS12 loss markedly attenuates electrically evoked dopamine release and accelerates dopamine reuptake—deficits that are reversed by KOR antagonism—demonstrating a functional disruption of KOR-dependent dopamine homeostasis within the ventral striatum of these knockout animals [34]. These findings implicate RGS12 as a key modulator of the established KOR-to-DAT regulatory axis [37,38] that shapes extracellular dopamine availability and psychostimulant sensitivity, with both processes closely tied to both mood regulation and attentional control. Subsequent studies demonstrated that *Rgs12* deletion also alters serotonergic transporter expression and function, suggesting broader monoaminergic involvement [36]. Collectively, these results position RGS12 as a pivotal integrator of both dopaminergic and serotonergic neurotransmission in the central nervous system.

Against this mechanistic backdrop derived from mouse genetics, the identification by Forstner et al. [35] of a rare familial BD-associated RGS12 variant (R59Q) in humans is particularly noteworthy. Surface plasmon resonance (SPR)-based in vitro binding studies indicate that the human R59Q variation within RGS12 impairs PDZ-domain-mediated protein–protein interactions essential for synaptic scaffolding [39]. Such a reduction in function could weaken RGS12’s inhibitory control over KOR-coupled G-protein signaling in the human brain, leading to excessive modulation of DAT and altered dopamine homeostasis.

In total, the recently characterized R59Q variation within the human RGS12 protein provides a biologically plausible molecular link between inherited BD risk and abnormal dopaminergic tone, thereby offering a mechanistic rationale for investigating whether RGS12 dysfunction influences psychostimulant responsiveness in BD patients with comorbid ADHD [40,41].

## 2. Results and Discussion

### 2.1. Multiple RGS12 Variations Seen in Psychiatric Disorders Including ADHD and BD

**Figure 1:**
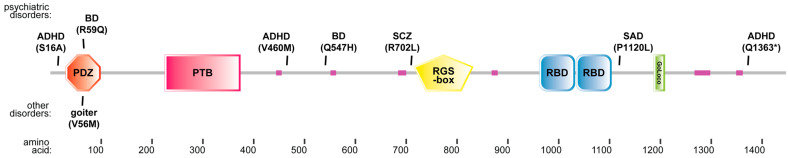
Multi-domain architecture of the GPCR negative regulator protein RGS12 and relative locations of human disease-associated missense or truncation variations. A domain map of the human RGS12 protein (UniProt O14924, also known as RGS12_HUMAN [1447 aa]) has been placed above an x-axis scale of amino acids. Known functional domains within RGS12 include a PDZ (PSD-95/disks-large/ZO-homology) domain [32], a PTB (phosphotyrosine-binding) domain [44], an RGS-box (also known as “Regulator of G protein Signaling” domain) [32], a tandem repeat of RBD (Ras-binding) domains [45], and a GoLoco (also known as “G_i/o_-Locomotion defects”) motif [46]; pink regions represent low-complexity polypeptide sequences predicted to lack secondary or higher-order structure. Above and below the domain map are the locations of non-synonymous substitutions to the consensus amino acid sequence (denoted by one-letter amino acid abbreviations) associated with the indicated mental health disorder or other pathological states. ADHD, attention-deficit/hyperactivity disorder; BD, bipolar disorder; SAD, schizoaffective disorder; SCZ, schizophrenia. (*) denotes truncation mutation (stop-codon).

To contextualize the functional relevance of RGS12 within neuropsychiatric disease, we conducted a comprehensive survey of reported human RGS12 variants across publicly available genomic and clinical databases. As of the time of writing this manuscript, there have been 241 missense variations observed within the human *RGS12* gene’s open-reading frame, as reported within NIH’s ClinVar database [42]: 5 in the PDZ domain, 29 in the PTB domain, 12 in the RGS-box, 21 in either the first or second RBD domain, and one truncation within the GoLoco motif, although none have yet been associated with a clinical pathology (Supplementary Table S1). In contrast, eight different RGS12 missense variations (not yet seen in ClinVar; Table S1) are reported in the current biomedical literature to be associated with disease states, the majority being psychiatric disorders, as summarized in Figure 1. Additionally, predicted loss-of-function variants of *RGS12* (i.e., frameshift and nonsense variants) are observed significantly less frequently (LOEUF = 0.696) than predicted in healthy individuals selected within the Genome Aggregation Database (gnomAD) [43], suggesting that *RGS12* is subject to selective constraint, with loss-of-function variants occurring less often than expected in the general population.

A parent-proband “trio” study of schizophrenia (SCZ), designed to detect de novo mutations rather than inherited variants, previously identified a non-synonymous variant (R702L) within RGS12 by whole-exome sequencing of 53 individuals with sporadic SCZ as well as their two non-affected parents [47]. An earlier SCZ-focused trio study [48] reported a de novo P1120L variant of RGS12 within a male proband diagnosed with schizoaffective disorder, depressed type. A study focused on ADHD susceptibility [49] identified a further three missense or truncating variants within the RGS12 protein (S16A, V460M, and Q1363* truncation) from whole-exomic sequencing of trios with sporadic ADHD. In identifying the R59Q variation associated with high-penetrance familial bipolar disorder [35], Forstner et al. also described an independently inherited Q547H variation within RGS12 (Figure 1) carried by three BD-affected members of a separate family among the 27 families analyzed. Considering the specific locations of all eight of these amino acid variations within the multi-domain architecture of the RGS12 scaffold protein, only the BD-associated R59Q variation (and the V56M variation in an autosomal dominant familial goiter [50]) resides within a domain or region with a well-defined functionality; specifically, the PDZ domain within the N-terminal ~100 amino acids of the *RGS12* open-reading frame (Figure 1).

### 2.2. Establishing a Reduction-of-Function Status to the PDZ Domain-Localized Variation

**Figure 2:**
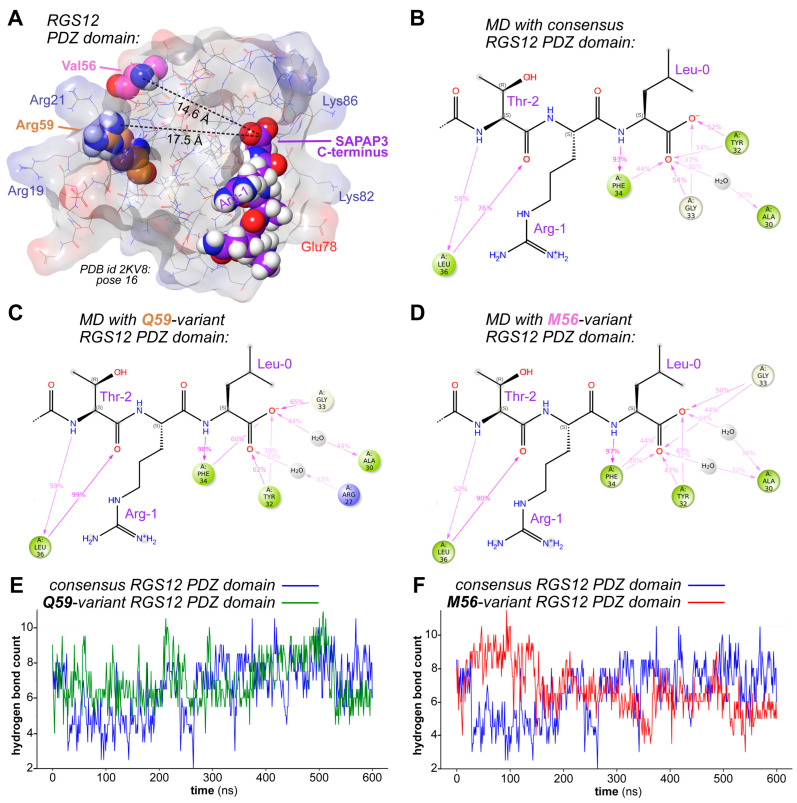
RGS12 PDZ domain amino acid variations associated with disease states are currently difficult to connect directly to their reduction-of-function status by computational approaches alone. (**A**), Three-dimensional representation of the predicted RGS12 PDZ domain/SAPAP3 C-terminal tail complex, based on our recent in silico docking [39] performed with the unpublished, solution NMR structural model of the unliganded RGS12 PDZ domain (Protein Data Bank id 2KV8). Highlighted are the distances between the carboxylic acid of the docked interacting peptide (**red** spheres, oxygen atoms; **purple** spheres, carbon) and the two positions reported to be different in separate families with high-penetrance bipolar disorder (glutamine [Q] instead of the consensus arginine at amino acid position 59; 17.5 angstroms) [35] and with autosomal dominant goiter (methionine [M] instead of the consensus valine at amino acid position 56; 14.6 angstroms) [50], respectively. (**B**–**D**), Summations of inter-atomic interaction occupancies over time (600 nanoseconds; **pink** arrows and percentages) during independent molecular dynamics simulations of the binding of the SAPAP3 C-terminal tail (final three amino acids of threonine–arginine–leucine shown) to indicate amino acid side chains (spheres) within the consensus RGS12 PDZ domain (panel (**B**)), the bipolar disease-associated variant RGS12 PDZ domain (panel (**C**)), and the familial goiter-associated variant RGS12 PDZ domain (panel (**D**)). (**E**,**F**), Total hydrogen bond counts between SAPAP3 C-terminal tail and PDZ domain atoms over 600 nanoseconds of simulation, as compared between consensus (**blue**) and bipolar-associated (**green**) RGS12 PDZ domains (panel (**E**)) or consensus and goiter-associated (**red**) RGS12 PDZ domains (panel (**F**)).

The PDZ domain, named after PSD-95 (postsynaptic density protein 95), Disks-large, and ZO-1 (zonula occludens-1), is a protein–protein interaction module commonly involved in signaling and scaffolding functions by virtue of its ability to bind the C-terminal tails of its protein partners [51,52]; the RGS12 PDZ domain is located between amino acids 30–99 (Table S1; https://www.uniprot.org/uniprotkb/O14924 [accessed on 30 November 2025]). Extensive knowledge now exists regarding the structure of PDZ domains and their features responsible for binding C-tail interactants (e.g., refs. [53,54]); however, we recently reported an inability to make a firm a priori prediction, via in silico structural modeling and molecular dynamics simulations [39], of the functional consequence of the glutamine substitution within the RGS12 PDZ domain associated with familial bipolar disorder (i.e., the R59Q variation). Similarly situated as the methionine substitution discovered in familial goiter [50], the glutamine substitution is predicted to occur at a distance far from the PDZ domain residues directly engaging C-tail ligands, as shown in Figure 2A rendered from our recently published structural model [39] of RGS12 interacting with the C-terminus of the protein DLGAP3 (also known as SAPAP3) [55]. This distance (>>10 angstroms) implies that any resultant change in C-tail binding affinity would be indirectly caused by allosteric remodeling within the domain rather than direct glutamine side-chain engagement of C-tail atoms [56,57]. Prior molecular dynamics simulations using AlphaFold2-generated RGS12 PDZ domain/SAPAP3 complexes [39], and newer simulations using our recent model of the RGS12/SAPAP3 interaction (Figure 2B–F), both fail to reveal substantial changes caused by amino acid substitutions at these positions to the hydrogen bonding network responsible for high affinity engagement of the C-tail ligand (e.g., Figure 2E,F). Nevertheless, our recent formal in vitro testing of the direct binding interaction using surface plasmon resonance indicates that the glutamine substitution at position 59 reduces the affinity of the RGS12 PDZ domain for the SAPAP3 C-tail over two-fold [39].

### 2.3. Reduction in Function Caused by the R59Q BD Variation Likely Leaves Methylphenidate Responsiveness Intact

**Figure 3:**
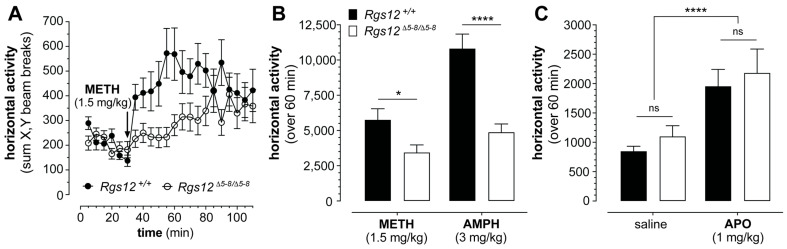
Hyperlocomotion upon treatment with methamphetamine (METH) or amphetamine (AMPH) is decreased in RGS12-deficient mice—an effect not caused by any apparent dopamine sensitivity change resulting from RGS12 loss. (**A**) Locomotor activity over time by *Rgs12*^Δ5–8/Δ5–8^ mice (open circles) versus wildtype littermate controls (closed circles), following 30 min acclimation to activity chambers and then methamphetamine administration (1.5 mg/kg ip, at a final volume of 10 mL/kg). Data plotted are the mean ± SEM (*n* = 11 for *Rgs12*-deficient mice, 13 for wildtype). (**B**) Methamphetamine (1.5 mg/kg ip)- and amphetamine (3 mg/kg ip)-induced hyperlocomotion as measured in *Rgs12*^Δ5–8/Δ5–8^ mice and wildtype littermate controls over 60 min after a 30 min acclimation to activity chambers then drug administration. Data for METH (1.5 mg/kg) are the mean ± SEM (*n* = 11–13 per genotype) as derived from panel (**A**) Data for AMPH (3 mg/kg) were previously obtained and reported in Gross et al. (2018) *J. Psychopharmacology (Sage Publications)* [33] and are represented here as the mean ± SEM (*n* = 20 for *Rgs12*-null mice, 23 for wildtype). Statistically significant differences by genotype were obtained by multiple unpaired *t*-tests: *, *p* < 0.05; ****, *p* < 0.00005. (**C**) Hyperlocomotion measured in *Rgs12*^Δ5–8/Δ5–8^ mice and wildtype littermate controls over 60 min after 30 min acclimation then treatment with control (saline) or the non-selective dopamine receptor agonist apomorphine (APO; 1 mg/kg ip). Data are the mean ± SEM (*n* = 10 for *Rgs12*-deficient mice, 14 for wildtype) and statistical significance evaluated by two-way ANOVA: ns, *p* > 0.05 (by genotype); ****, *p* < 0.0001 (by treatment).

What could the two-fold reduction in RGS12 PDZ domain binding affinity mean to those harboring variations like the R59Q substitution in familial BD? To further investigate possible functional consequences, we revisited the responsiveness of RGS12-deficient mice to acute psychostimulant administration. We previously reported that RGS12-deficient mice exhibit significantly reduced hyperlocomotor responses to dopamine transporter (DAT)-dependent psychostimulants, including cocaine and amphetamine, given increased dopamine uptake and reduced extracellular dopamine levels in the ventral striatum engendered by RGS12 deficiency [33,34]. We recently extended this observation to the psychostimulant methamphetamine (Figure 3), which, like cocaine and amphetamine, ultimately increases extracellular dopamine, albeit via different monoamine transporter affinities [58] and potentially via different signal transduction timing than cocaine and AMPH (e.g., ref. [59]). Reduced hyperlocomotion by RGS12-deficient mice to these DAT-dependent psychostimulants does not appear to result from decreased postsynaptic receptor sensitivity to dopamine, as the induction of hyperlocomotion upon apomorphine administration (a non-selective D1/D2 dopamine receptor agonist) appears unchanged by mouse genotype (Figure 3C); we had previously dismissed [33] dopamine sensitivity change as a plausible cause of reduced psychostimulant-induced hyperlocomotion upon RGS12 loss via the application of the D1/D5-selective dopamine receptor agonist SKF-81297 [60,61].

**Figure 4:**
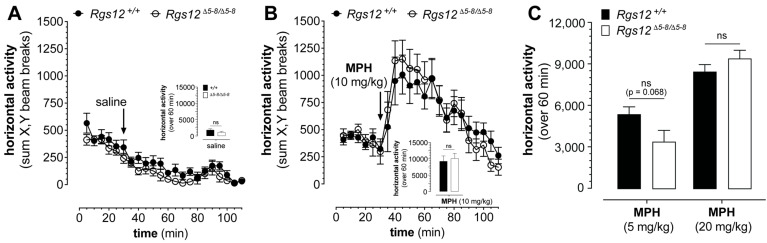
Methylphenidate (MPH) elicits hyperlocomotion equivalently in wildtype and RGS12-deficient mice. (**A**). *Rgs12*^Δ5–8/Δ5–8^ mice (open circles) versus wildtype littermate controls (closed circles), following 30 min acclimation to activity chambers and then administration of saline (panel (**A**)) or methylphenidate (10 mg/kg, ip; panel (**B**)). Data plotted are the mean ± SEM (*n* = 7 for *Rgs12*-deficient mice, 8 for wildtype). *Inset for both panels*: bar graphs of locomotion summed over 60 min post-acclimation and drug administration; statistical significance by genotype evaluated by multiple unpaired *t*-tests: ns, *p* >> 0.05. (**C**) Hyperlocomotion induced by separate 5 mg/kg or 20 mg/kg doses (ip) of methylphenidate, as measured in *Rgs12*^Δ5–8/Δ5–8^ mice and wildtype littermate controls over 60 min after a 30 min acclimation to activity chambers then drug administration. Data for 5 mg/kg MPH treatment are the mean ± SEM (*n* = 8 for *Rgs12*-null mice, 7 for wildtype); data for 20 mg/kg MPH treatment are the mean ± SEM (*n* = 12 for each genotype). Statistical significance by genotype was evaluated by multiple unpaired *t*-tests: ns, *p* > 0.05.

What of methylphenidate in this context, especially given existing treatment heuristics that methylphenidate for BD/ADHD comorbidity is often first-line before considering amphetamine [8,10,62] (and with methamphetamine considered too likely to induce manic episodes given its relatively higher potency and fast-acting onset [63,64])? Compared to mock injection with saline, treatment of RGS12-deficient mice with methylphenidate is seen to elicit hyperlocomotion to the same degree as in wildtype littermate control mice (e.g., Figure 4A vs. Figure 4B); three different doses of methylphenidate (5, 10, and 20 mg/kg body weight) elicited equivalent hyperlocomotive responses between the two genotypes, without any statistically significant difference observed in open-field transit over 60 min for each dose (Figure 4B,C). These new findings effectively negate any assumption, arising from our prior RGS12 work and the R59Q variant discovery, that RGS12 dysfunction constitutes a contraindication for methylphenidate use in BD/ADHD comorbidity. Likely mechanistic discriminators driving reduced responses to cocaine [33], amphetamine [33,34], and methamphetamine (Figure 3B) versus our observation of an equivalent methylphenidate response (Figure 4) in the presence of RGS12 deficiency is that methylphenidate does not directly promote neurotransmitter release like amphetamine and methamphetamine [65], and is far less serotonergic than cocaine in the context of monoamine transporter inhibition (e.g., ref. [58]). Unlike amphetamines, which induce dopamine release via a combination of DAT reverse transport [66], vesicular monoamine transporter 2 (VMAT2) inhibition [67,68], and TAAR1 receptor signaling [69], MPH acts primarily as a norepinephrine–dopamine reuptake inhibitor (NDRI) with greater monoamine transporter selectivity and more potent NET inhibition relative to cocaine [70]. While we have reported that RGS12 deficiency in mice leads to changes in the expression levels and functional activities of DAT [33,34] and SERT [36], we currently have no empirical evidence suggesting aberrant NET activity or norepinephrine dynamics upon loss of RGS12 function.

### 2.4. Study Limitations and Future Directions

Several limitations of this study warrant consideration. First, although *Rgs12*-deficient mice provide a robust genetic model for exploring RGS12 function in dopaminergic regulation, the frequency and clinical penetrance of RGS12 reduction-of-function variants, such as R59Q, within broader bipolar disorder (BD) populations, and particularly within BD patients comorbid for ADHD, remain undetermined. Future genomic analyses of large, phenotypically stratified BD/ADHD cohorts will be essential to define the population-level relevance of such variants. Second, our behavioral assays focused primarily on psychostimulant-evoked locomotor activity, which serves as a reliable proxy for dopaminergic sensitivity but does not capture potential subtler cognitive or affective phenotypes that may accompany RGS12 dysfunction. Future studies employing electrophysiologic, voltametric, or circuit-level imaging approaches in awake, behaving animals could further clarify the precise neural consequences of RGS12 loss on dopamine and norepinephrine signaling. Finally, translational work incorporating induced pluripotent stem cell (iPSC)-derived neuronal models or humanized knock-in mice carrying the R59Q variation to the RGS12 PDZ domain would provide a direct bridge between these preclinical observations and the human condition.

## 3. Materials and Methods

### 3.1. Database Searches

Searches were performed in PubMed (https://pubmed.ncbi.nlm.nih.gov [accessed on 30 November 2025]), ClinVar (https://www.ncbi.nlm.nih.gov/clinvar [accessed on 30 November 2025]), and the Genome Aggregation Database (gnomAD v.4.1.0 [accessed on 30 November 2025]), using the NCBI Gene identifier 6002 for human RGS12.

### 3.2. MD Simulations of PDZ Mutations

600 nanosecond molecular dynamics (MD) simulation trajectories of SAPAP3-liganded RGS12 PDZ domains were performed as previously published [39], after replacing the AlphaFold2-generated PDZ domain structural model with that of the best-performing low-energy NMR-derived structural model (PDZ id 2KV8 pose 16) as ranked by favorable docking scores from Schrödinger’s GLIDE SPpep and XP docking (software suite version 2024-4) [40,41] of C-terminal pentameric peptides from SAPAP3 and two other candidate RGS12 interactors: CXCR2 and MEK2 [39].

### 3.3. Locomotion Studies

Mouse strain maintenance and behavioral testing for the present study were both performed as previously described [33,34,36]; key methodological details are briefly summarized here.

#### 3.3.1. Animals

*Rgs12*^Δ5–8/Δ5–8^ mice were generated on a C57BL/6J background as previously described [33,34,36]. Male and female littermates were produced by heterozygous crosses and group-housed under controlled temperature, humidity, and a 12 h light/12 h dark cycle (lights on at 06:00 h) with food and water ad libitum. Behavioral testing occurred during the light phase using mice 8–12 weeks of age (typically 8–10 weeks). All procedures conformed to the NIH guidelines for the care and use of laboratory animals and approved by the West Virginia University Institutional Animal Care and Use Committee. All behavioral cohorts were drug-naïve, and each mouse participated in only one drug-based assay.

#### 3.3.2. Drugs and Dosing Rationale

All drugs were purchased from Sigma-Aldrich (St. Louis, MO, USA) or Tocris (Minneapolis, MN, USA). Each dose and route was selected based on prior literature demonstrating reliable locomotor activation without neurotoxicity in C57BL/6 mice. Psychostimulants were prepared freshly in sterile 0.9% NaCl (saline) and administered intraperitoneally (ip) in a final volume of 10 mL/kg body weight. The chosen doses replicate those validated in our earlier work [33,34,36] and standard in C57BL/6 mouse psychostimulant paradigms:

- **d-amphetamine hemisulfate**, 1–5 mg/kg (ip), produces robust hyperlocomotion with maximal effect near 3 mg/kg;
- **methamphetamine hydrochloride**, 1.5 mg/kg (ip), elicits a comparable dopamine-dependent hyperlocomotor response used here to directly compare with amphetamine;
- **methylphenidate hydrochloride**, 5–20 mg/kg (ip), covers the established dose–response range for norepinephrine–dopamine reuptake inhibition in rodents;
- **apomorphine**, 1 mg/kg (ip), a non-selective D_1_/D_2_ dopamine receptor agonist, serves as a positive control for postsynaptic dopamine responsiveness.

#### 3.3.3. Behavior Testing

Open-field activity was measured using 16 × 16 photobeam activity system (PAS) chambers (San Diego Instruments, San Diego, CA, USA; enclosure 16 × 16 × 15 inches) under standard environmental conditions. Horizontal locomotion was recorded as total x- and y-beam breaks in 5 min bins. To minimize novelty-induced activity, mice were acclimated to the testing room for ≥1 h and to the chamber for 150 min on Day 1. On Day 2, mice were placed in chambers for 30 min, administered saline (ip, 10 mL/kg), and monitored for ~110 min. On Day 3, mice were again acclimated for 30 min, then received psychostimulant or vehicle injections as above, and locomotor activity was recorded for 60–110 min depending on the compound. Each chamber was cleaned between subjects with water-moistened cloths.

#### 3.3.4. Data Analysis

Data are presented as mean ± SEM and analyzed using GraphPad Prism 10. Between-genotype differences in locomotor responses were evaluated by two-way ANOVA or multiple unpaired Student *t*-tests as appropriate, consistent with prior statistical treatments [33,34,36]. Sample sizes per group ranged from 7 to 23 mice for amphetamine/cocaine trials and 7–13 mice for methamphetamine or methylphenidate trials, as indicated in figure legends.

## 4. Conclusions

Preserved methylphenidate-evoked hyperlocomotion in RGS12-deficient mice indicates that stimulant efficacy can remain intact despite RGS12 loss-of-function. Genetic testing for RGS12 functional variants should not be used as a basis for excluding methylphenidate to treat ADHD in mood-stabilized BD patients.

## Data Availability

The raw data supporting the conclusions of this article will be made available by the authors via e-mail upon reasonable request.

## Supplementary Materials

The following supporting information can be downloaded at https://www.mdpi.com/article/10.3390/ijms262411993/s1.
